# Clinical Benefits of an Adherence Monitoring Program in the Management of Secondary Hyperparathyroidism with Cinacalcet: Results of a Prospective Randomized Controlled Study

**DOI:** 10.1155/2013/104892

**Published:** 2013-07-18

**Authors:** Valentina Forni Ogna, Menno Pruijm, Carole Zweiacker, Grégoire Wuerzner, Eric Tousset, Michel Burnier

**Affiliations:** ^1^Division of Nephrology, Department of Medicine, Centre Hospitalier Universitaire Vaudois, Rue du Bugnon 17, 1011 Lausanne, Switzerland; ^2^AARDEX Group Ltd., Rue des Cyclistes Frontiére 24, B4600 Visé, Belgium

## Abstract

*Background/Aims*. One of the causes of uncontrolled secondary hyperparathyroidism (sHPT) is patient's poor drug adherence. We evaluated the clinical benefits of an integrated care approach on the control of sHPT by cinacalcet. *Methods*. Prospective, randomized, controlled, multicenter, open-label study. Fifty hemodialysis patients on a stable dose of cinacalcet were randomized to an integrated care approach (IC) or usual care approach (UC). In the IC group, cinacalcet adherence was monitored using an electronic system. Results were discussed with the patients in motivational interviews, and drug prescription adapted accordingly. In the UC group, drug adherence was monitored, but results were not available. *Results.* At six months, 84% of patients in the IC group achieved recommended iPTH targets versus 55% in the UC group (*P* = 0.04). The mean cinacalcet taking adherence improved by 10.8% in the IC group and declined by 5.3% in the UC group (*P* = 0.02). Concomitantly, the mean dose of cinacalcet was reduced by 7.2 mg/day in the IC group and increased by 6.4 mg/day in the UC group (*P* = 0.03). *Conclusions.* The use of a drug adherence monitoring program in the management of sHPT in hemodialysis patients receiving cinacalcet improves drug adherence and iPTH control and allows a reduction in the dose of cinacalcet.

## 1. Introduction 

Poor adherence is an important problem in the dialysis population, where patients should not only adhere to medication intake but also to strict dialysis hours and complicated dietary recommendations [1]. Depending on the definition used for adherence (previously called compliance), between 50% and 85% of dialysis patients may be considered nonadherent [2]. Low adherence to drugs used in the treatment of renal bone disorders is particularly worrisome, since high levels of intact parathyroid hormone (iPTH), elevated phosphorus (PO_4_), and Ca × PO_4_ product have been associated with increased morbidity and mortality rates [3–6]. Although the lack of effectiveness of drug therapy may also play a role, adherence is an important issue since the average patient takes six to ten pills per day to control their calcium phosphate product [7], and numerous studies have demonstrated that adherence is inversely related to the number of pills taken daily [8].

The calcimimetic cinacalcet is an effective drug for the treatment of secondary hyperparathyroidism (sHPT) in dialysis patients [9, 10]. It is actually unclear whether a lack of response to a therapeutic dose of cinacalcet is the result of resistance to the drug or of poor adherence. Distinguishing these possibilities is clinically relevant, because increasing the dose in a nonadherent patient will only increase the costs of the treatment, without improving the clinical results. In this respect, in a retrospective cohort study including 4923 patients in the USA, greater cinacalcet adherence was associated with inpatient cost savings of $4000–$8900/patient/year [11].

An effective way to monitor adherence to medication intake is the “Medication Events Monitoring System” (MEMS). This electronic monitoring system records each time (date and hour) the pillbox containing the drug under investigation is opened. Today this is considered as the most sensitive and valid method to measure medication adherence [12]. We have previously shown in a pilot study [13] that measuring the adherence to cinacalcet and phosphate binders allows the detection of nonadherence and thereby improves the control of sHPT in hemodialysis patients with suspected nonadherence. However, as the study was uncontrolled, it remained unclear whether this improvement was simply the effect of introducing the electronic monitoring system, or whether it was the result of the discussions of adherence data which took place between physician, patient, and pharmacist. 

The main objective of this prospective randomized controlled study was to assess whether an integrated care (IC) approach, in which adherence data are integrated in the decisional process, leads to improved therapeutic control of sHPT and higher percentages of bone metabolism targets as compared to a usual care (UC) approach, in which biological values represent the main stem of the decisional process. Moreover, we hypothesized that the bone metabolism targets would be reached at a lower dose of cinacalcet in the IC group as compared to the UC group.

## 2. Subjects and Methods

This was a prospective randomized, multicenter open-label study performed in nine dialysis facilities in Switzerland. Adult patients on maintenance hemodialysis (MHD) since more than three months, treated with a daily dose of cinacalcet of ≥30 mg for at least one month and iPTH values in or above target according to the treating nephrologists, were eligible for inclusion. Patients with intolerance to cinacalcet, previous or planned parathyroidectomy for suspected tertiary hyperparathyroidism, or patients with hypocalcaemia (serum calcium < 7.48 mg/dL (< 1.87 mmol/L)) were excluded. Other exclusion criteria were the inability to understand the protocol, mental diseases, or a short-life expectancy (less than six months). The study was carried out under good clinical practice, according to the principles of the Declaration of Helsinki. Written informed consent was obtained from each patient. The study was approved by all regional ethical committees. The study was registered as ClinicalTrials.gov (Nr.268/09). 

Eligible patients were randomly assigned 1 : 1 to the IC or UC groups. A central randomization system was used, assigning participants within centers in blocks of four. After inclusion, adherence to cinacalcet was monitored using the MEMS system (MEMS SmartCap, Medication Event Monitoring System, Aardex Group, Ltd., Sion, Switzerland) for six months, followed by an observation period of three months without monitoring in each group. In the intervention group (IC group), adherence data were available for therapeutic decisions, whereas in the UC group adherence data were blinded until the end of the study. In the IC group, drug adherence results were discussed with the patients by the treating nephrologists in semistructured motivational interviews at intervals of two months. The first motivational interviews based on MEMS results took place after two months in the IC group; they were repeated every two months thereafter. No adherence data were available in the UC group throughout the study.

**Figure 1:**
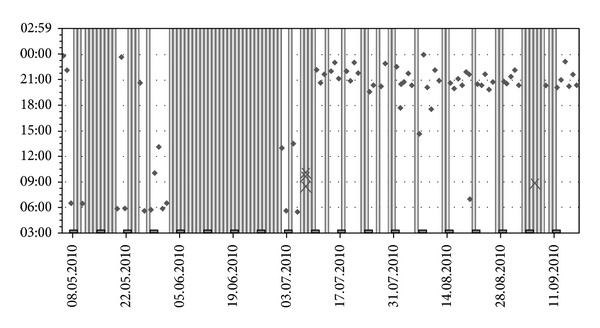
Example of MEMS graphical report (once a day prescription). Note: points correspond to an MEMS-box opening, and grey bars correspond to days without opening.

During the interviews, patients were asked to describe their experience and complications with medication intake. Drug adherence results were discussed through the help of the MEMS graphical report, which allowed the direct visualization of date and hour of the consecutive MEMS-box openings (see Figure 1). Potential barriers to adherence were identified, strategies to overcome the barriers were generated with the patient, and an individualized adherence plan was elaborated. At each subsequent meeting, the interventionist evaluated whether the strategies had been implemented and generated alternative strategies if necessary. Of note, none of the main authors of this study were involved in drug prescription, nor were any of the treating physicians involved in the statistical analysis of the results of the study.

In the UC group, therapeutic decisions were based on blood chemistry only. Blood samples for iPTH, phosphorus, and total calcium were obtained at baseline and at two, four, six, and nine months. Blood analyses were performed in each local center.

iPTH, phosphorus, and calcium targets, as well as the prescription of dialysis treatments and additional drugs were left to the discretion of the treating nephrologists. Importantly, in both groups physicians were asked to define their target values of iPTH, phosphorus, and calcium in their center before starting the protocol. Cinacalcet dose changes were performed by the treating nephrologists at two-month intervals between baseline and six months and at their discretion between six and nine months. Cinacalcet was conditioned in the electronic pillbox every two months by center collaborators, according to Good Pharmacist Practices guidelines. Patients were instructed to open the pillbox when it was time to take cinacalcet, to remove the prescribed dose, and to close the package directly thereafter.

In the IC group, dosing history data were downloaded from MEMS and discussed with the treating nephrologist at the end of each two-month interval. For the UC group, dosing history data were downloaded at the end of each two-month interval; neither the patient nor the treating nephrologists had access to the adherence results until the end of the study (blinding). Before the start of the study, physicians and patients were asked to predict patient's adherence status using a categorical scale from 1 (very poor, ≤50% prescribed doses taken), 2 (50–80% prescribed doses taken), and 3 (85–95% prescribed doses taken) to 4 (excellent, ≥95% prescribed dose taken). 

Endpoints of the study were the mean dose of cinacalcet at six months needed to achieve the iPTH targets values, the absolute values and changes in iPTH values at six months from baseline, changes in cinacalcet taking adherence at six months from baseline, absolute and relative change in cinacalcet doses at six months from baseline, and the percentage of patients on iPTH target at six months. The parameter used to assess adherence was the *taking adherence*, defined as the ratio between the number of dose taken and the number of dose prescribed. 

For a complete evaluation of the practice patterns in sHPT management, the usage of vitamin D sterols (oral calcitriol, oral and IV paricalcitol) and phosphate binders was assessed at two-month intervals until the end of the study. Safety and tolerability were assessed in terms of the incidence of adverse drug reactions (ADR), serious ADRs, and deaths. An ADR was defined as an adverse event that the investigator considered to be attributed to the use of cinacalcet.

Continuous variables were summarized as means (standard deviation) for normally distributed variables or median (interquartile range (iqr)) for nonnormally distributed continuous variables. Chi-square test was used to compare categorical data. Student's *t*-test was used for normally distributed continuous variables, and Wilcoxon rank-sum test was used to compare nonnormally distributed continuous variables between the two groups; Wilcoxon signed-rank test was used to compare matched pairs of non-normally distributed continuous variables within groups. A two-sided *P* value < 0.05 was considered to be significant. Analyses were performed using STATA software version 11.0.

## 3. Results

**Figure 2:**
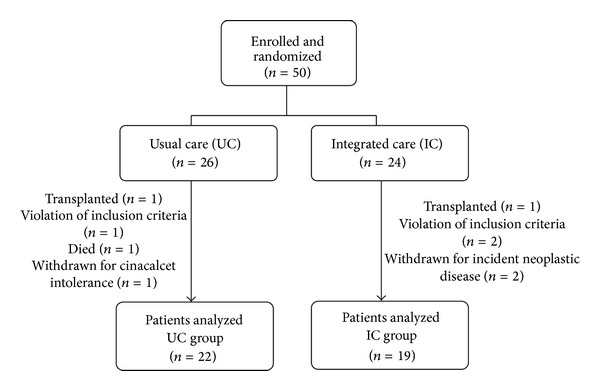
Schematic representation of patients randomization and followup.

The study took place between January 2010 and April 2012. The study flow diagram is shown in Figure 2.

Out of the 50 patients initially enrolled and included in the study (*n* = 24 in the IC group, *n* = 26 in the UC group), five patients in the IC group and four in the UC group did not complete the study period, for several reasons: kidney transplantation (*n* = 2), death (*n* = 1), incident neoplastic disease (*n* = 2), cinacalcet-related de novo gastrointestinal side effects (*n* = 1), and violation of the predefined exclusion criteria of previous or planned parathyroidectomy for suspected tertiary hyperparathyroidism (*n* = 3). Only patients who completed the six-month study period were considered in the final analysis.

Baseline characteristics of the UC group (*n* = 22) and the IC group (*n* = 19) were comparable with regard to age, sex, dialysis time, biologic parameters, and prescription of drugs (Table 1), with the exception of a higher paricalcitol dose in the IC group at baseline (*P* < 0.05).

### 3.1. Control of sHPT

**Figure 3:**
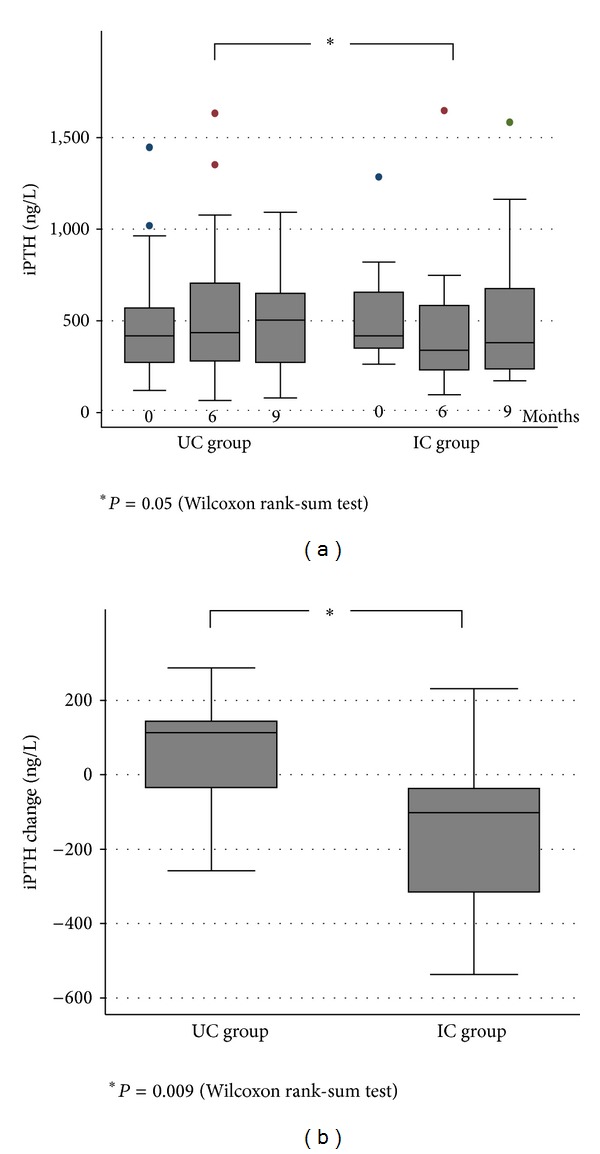
Absolute intact parathyroid hormone (iPTH) values (a) and iPTH mean changes (b). Note: conversion factors for units: iPTH in ng/L to pmol/L, divided by 9.43.

The median (iqr) iPTH value decreased in the IC group from 417 ng/L (iqr: 352; 622) at baseline to 339 ng/L (236; 529) after six months (*P* = 0.03). In the UC group, no significant change was observed: median (iqr) iPTH 419 ng/L (275; 548) at baseline and 436 ng/L (288; 682) at six months (*P* = 0.1) (Figure 3(a)). Hence, the median (iqr) change in iPTH was negative in the IC group: −94.3 ng/L (−282.6; −27.7) and positive in the UC group: +113.6 ng/L (−26.2; 145.1) (*P* = 0.009) (Figure 3(b)). Of note, after discontinuation of the MEMS monitoring (months from six to nine), median (iqr) iPTH values rose slightly in both groups: +16.0 ng/L (−163.3; +197.1) in the UC group (*P* = 1.0) and +50.0 ng/L (−85.8; +304.1) in the IC group (*P* = 0.2) (Figure 3(a)). The other biological parameters of phosphorus and calcium metabolism, namely, serum Ca, PO_4_, and Ca × PO_4_ product, did not differ between the groups, nor were any changes observed throughout the study. At 6 months, 84% of patients in the IC group had iPTH levels within the KDIGO guidelines, as compared to 55% of the UC group (*P* = 0.04). Only 42% of the IC group and 23% of patients of the UC group had iPTH levels in the KDOQI range (*P* = 0.2). 68% of patients in the IC group and 50% in the UC group achieved the predefined nephrologists targets (*P* = 0.2). 

### 3.2. Prescription of Cinacalcet

**Figure 4:**
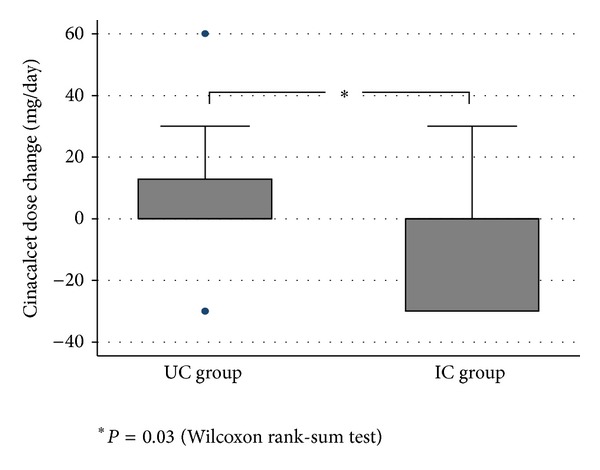
Change in cinacalcet dose over six-month MEMS monitoring.

The mean (sd) cinacalcet dose could be reduced from 42.0 (20.8) to 34.7 (26.5) mg/day in the IC group, corresponding to a mean (sd) change of −7.2 (19.8) mg/day (−17%), whereas in the UC group there was a dose increase from 31.6 (13.4) to 38.0 (24.6) mg/day, corresponding to a mean (sd) change of +6.4 (19.9) mg/day (+20%) (*P* = 0.03). Hence, the relative difference in percentage of dose change between the two groups was 37% (Figure 4). Considering other concomitant treatments, no difference between groups was noted throughout the study. 

### 3.3. Drug Adherence

**Figure 5:**
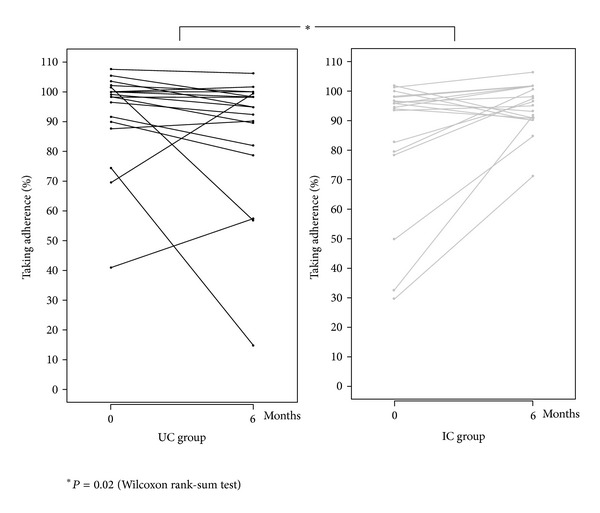
Change in individual taking adherence over six-month MEMS monitoring.

During the first two months of MEMS monitoring, the mean (min-max) percentage of taking adherence was 84.4% (29.8–101.8) in the IC group and 93.7% (41.0; 107.7) in the UC group (*P* = 0.1). At the end (last two months of monitoring) of the six-month MEMS monitoring period, the mean (sd) intake increased by 10.3% (18.6) in the IC group. In contrast, it decreased by 5.5% (19.8) in the UC group (*P* = 0.02) (Figure 5). We found a good concordance between physician predicted adherence and measured patient adherence at baseline, when fixing a cutoff at 80%: 87.5% of patients (7 out of 8) with taking adherence at baseline <80% and 75.8% (25 out of 33) with taking adherence >80% were correctly identified by the physician. Only 62.5% (5 out of 8) patients with taking adherence <80% and 81.8% (27 out of 33) of patients with adherence >80% correctly predicted their results before start of adherence monitoring.

In subgroups analysis, those patients with lower (≤90%) taking adherence at baseline benefited most from the IC approach: their mean (sd) change in taking adherence was +31.5% in the IC group versus −4.5% in the UC group (*P* = 0.04). For patients with taking adherence at baseline >90%, no difference was observed between the two approaches. Consistently with the previous observation, patients with taking adherence at baseline ≤90% showed a significant biological response to the IC approach, with a median (iqr) iPTH change at six months from baseline of −101.8 ng/L (−424; −78.6) versus +114.1 ng/L (−96.0; +614.0) in the UC group (*P* = 0.03). 

### 3.4. Tolerability and Safety

Adverse events were reported in 0.7% of patients, namely, gastrointestinal disturbances (three cases), necessitating drug suspension in one case. Hypocalcemia (serum calcium < 7.48 mg/dL, < 1.87 mmol/L) was observed in 29 over 369 measures (7.9%), none of which were symptomatic. Serious AEs occurred in two patients: one died (sudden cardiac death unrelated to cinacalcet intake); the second patient was prematurely withdrawn from the study because of incident neoplastic disease necessitating hospitalization. 

## 4. Discussion

Taken together, the results of the present study confirm that an integrated care (IC) approach significantly improves the control of secondary hyperparathyroidism (sHPT) in dialysis patients: the IC approach improves the adherence to cinacalcet, enables to reduce the cinacalcet dose without changing the prescription of phosphate binders and vitamin D derivatives, and increases the number of patients reaching iPTH targets. However, the positive effects of the intervention were maintained only during the monitoring and had vanished three months after the interruption of the drug adherence monitoring, suggesting that the intervention should be of longer duration. 

To the best of our knowledge, this is the first prospective randomized study in haemodialysis patients that analyzed the impact of integrating drug adherence data obtained by electronic pillbox monitoring on the control of sHPT. One common criticism to the use of electronic monitoring of drug adherence in interventional studies is that the introduction of the device per se enhances drug adherence and hence improves clinical results. Our controlled study demonstrates that simply introducing electronic monitoring, without disclosing adherence results to physicians and patients, is not enough to improve adherence and sHPT control and does not improve the clinical targets. As such, the existence of an “adherence increasing effect” due to the use of an electronic monitoring system alone without feedback to the patients is not supported by our data. Indeed, only an integrated care approach, combining the interpretation of the unique patient's data by a trained physician and the periodical feedback to the patient, led to a therapeutic benefit. This further emphasizes the importance of the quality of the physician/patient interaction in improving drug adherence. With the IC approach we actually counteracted and reversed the trend of drug adherence to decline over time as observed in our control group and in previous observational studies [14, 15]. The high sensitivity of iPTH control to cinacalcet adherence is not surprising, when considering the well-established short half-life of cinacalcet and its immediate effect on iPTH level [16].

One might argue that the changes in iPTH levels, reduction in cinacalcet dose, and improvement in adherence were relatively modest. This is most likely due to the fact that the inclusion of patients was not restricted to those with a low drug adherence at baseline. Thus, in order to simulate “real-life practice,” even patients with a good adherence at baseline were enrolled. Moreover, drug adherence monitoring was only performed during six months. It is, however, well recognized that drug adherence decreases progressively with time in many diseases [14]. In this respect, it is important to note that patients with estimated baseline taking adherence below 90% (26.8% of all patients) mostly benefited from the integrated care approach. These data indicate that, if this approach would be introduced in general practice, it is crucial to carefully select patients for monitoring in order to avoid excessive expenses and labour forces. In this context, we propose the use of a step-up approach. Electronic monitoring of drug adherence should be considered in all patients with suspected poor adherence or patients needing very high doses of cinacalcet to control iPTH. If a low drug adherence is confirmed by the monitoring or if clinical values improve significantly during the monitoring, the IC approach could be proposed in order to sustain drug adherence in the long run. It is clear that the clinical effectiveness of such an approach needs to be validated in a prospective study.

The loss of effect after the interruption of the IC approach is classical and has been observed in previous studies assessing drug adherence in other pathologies [12]. It indicates a lack of long-term effect of the motivational teaching. This might be partly related to the short duration of our intervention (six months). Our findings are also in line with the statements that adherence is a dynamic parameter [14] and that continuous efforts are needed to ascertain adherence over time. Further study is needed to assess the duration of the IC approach needed to obtain a definite change in behaviour. Nevertheless, the improvement of clinical parameters during the monitoring provides important information for physicians, as it indicates what clinical results can be obtained when the patient is taking his/her treatment correctly. Thus, when clinical parameters worsen again, physicians should probably intervene on drug adherence rather than increase the drug doses.

Two other points deserve attention. First, mean baseline adherence in our study was high and superior to the threshold of 80% that arbitrarily defines good adherence [17]. Considering the complex drug regimens (six–ten pills/day) imposed to dialysis patients and the known inverse relationship between drug adherence and the number of daily tablets to be taken [7, 18], we had expected to find lower drug adherence results. However, adherence results were similar to those observed in kidney transplant patients on immunosuppressive drugs [18] and hypercholesterolemic patients treated with atorvastatin [19]. Once again, the short duration of our study can probably account for these good results as drug adherence tends to decrease after six months. 

Second, physicians' ability to recognize patients with poor adherence was good, with over 80% of patients with taking adherence <80% being correctly identified before adherence monitoring started. Conversely, only 62.5% of patients with poor adherence (<80%) acknowledged adherence problems before the start of the study. This is in contrast with previous data from other disciplines [12]. A possible explanation might be that nephrologists are better able to predict patient adherence, thanks to the unique relationship with their patients, consisting (in general) of several patient-physician contacts per week. 

The main limitation of this multicenter study design is the lack of a central laboratory; all biochemical markers of the bone metabolism were measured locally. In order to minimize heterogeneity of iPTH levels, exclusively second-generation PTH assays measuring the intact 1–84 PTH were used in all centers, and the same assay was used in the same patient throughout the study. However, we cannot exclude residual variance in laboratory parameters.

Contamination between study arms might have occurred, due to the fact that the same physician took care of both study groups. However, physicians were blinded to drug adherence results of the UC group until study end. They therefore lacked the most important objective tool to evaluate drug adherence and elaborate a corrective intervention. Furthermore, an effort was made to standardize motivational interviews, in order to homogenize the intervention between study centers. 

In conclusion, the use of a drug adherence monitoring-based interventional approach in hemodialysis patients receiving cinacalcet for sHPT enabled to unmask and improve drug adherence problems and to transiently achieve better iPTH control at a lower dose of cinacalcet. Patients with poor adherence at baseline mostly benefitted from the integrated care approach, suggesting that in this patient group the adherence monitoring-based interventional approach should be proposed before increasing cinacalcet dose. 

## Figures and Tables

**Table 1:** Baseline clin1ical characteristics of patients who completed the study.

	UC group	IC group	*P* value
*N*	22	19	
Age (years)	61.3 (9.8)	59.1 (15.6)	0.6
Men (%)	59	79	0.2
BMI (kg/m^2^)	26.5 (3.5)	26.4 (4.1)	0.7
Previous transplant (%)	33	16	0.4
Time on dialysis (months)	50 (48)	50 (45)	0.6
Dialysis time (hours/week)	11.4 (0.9)	11.7 (0.5)	0.3
Calcium dialysate (mmol/L)	1.59 (0.91)	1.53 (0.84)	0.8
iPTH baseline	419 (275; 548)	417 (352; 622)	0.5
Cinacalcet dose (mg/d)	31.6 (13.4)	42.0 (20.8)	0.1
Cinacalcet taking adherence	93.7 (3.3)	84.4 (5.4)	0.1
PO_4_-chelators (*n*)	1.1 (0.6)	1.2 (1.2)	0.7
Active Vit. D3 (calcitriol) (mcg/week)	1 (0.3)	1.3 (0.5)	0.7
Active Vit. D3 (paricalcitol) (mcg/week)	0	6 (3)	<0.05
Inactive Vit. D3 (UI/week)	2179 (4078)	1597 (3351)	0.5

Values are expressed as mean (standard deviations) or median (25th; 75th percentile) as appropriate.
